# Patterns of Care and Outcome Analysis of Nasopharyngeal Carcinoma: An Indonesian Single Institution Study

**DOI:** 10.31557/APJCP.2020.21.5.1481

**Published:** 2020-05

**Authors:** Hamida Hayati Faisal, Nobuteru Kubo, Endang Nuryadi, Joedo Prihartono, Tubagus Djumhana Atmakusuma, Lisnawati Rachmadi, Takahiro Oike, Takashi Nakano, Soehartati A Gondhowiardjo, Marlinda Adham

**Affiliations:** 1 *Department of Ear, Nose and Throat, Head and Neck Surgery, Dr. Cipto Mangunkusumo National General Hospital, Faculty of Medicine Universitas Indonesia, Jakarta, Indonesia. *; 2 *Department of Radiation Oncology, Gunma University Graduate School of Medicine, Gunma, Japan. *; 3 *Department of Radiation Oncology, Dr. Cipto Mangunkusumo National General Hospital, Faculty of Medicine Universitas Indonesia, Jakarta, Indonesia. *; 4 *Department of Community Medicine, Faculty of Medicine Universitas Indonesia, Jakarta, Indonesia. *; 5 *Department of Internal Medicine, Hematology and Medical Oncology Division, Dr. Cipto Mangunkusumo National General Hospital, Faculty of Medicine Universitas Indonesia, Jakarta, Indonesia. *; 6 *Department of Pathological Anatomy, Dr. Cipto Mangunkusumo National General Hospital, Faculty of Medicine Universitas Indonesia, Jakarta, Indonesia. *

**Keywords:** Nasopharyngeal carcinoma, radiation therapy, patterns of care, treatment outcome, Indonesia

## Abstract

**Background:**

Nasopharyngeal cancer is endemic to Southeast Asia. However, there is limited clinical evidence of nasopharyngeal cancer in Indonesia, which has the largest population in Southeast Asia.

**Methods:**

Patterns of care and treatment outcomes in 428 patients with newly-diagnosed and pathologically-confirmed nasopharyngeal cancer were retrospectively analyzed.

**Results:**

Concurrent chemo-radiotherapy (CCRT) was the first-line treatment for stages I–IVB diseases. The 2-year overall survival (OS) of all patients were 100.0%, 100.0%, 93.8%, 86.2%, 82.9%, and 62.4% for stages I, II, III, IVA, IVB, and IVC, respectively. The 2-year OS of CCRT-treated patients were 100.0%, 100.0%, 92.6%, 82.4%, and 78.3% for stages I, II, III, IVA, and IVB, respectively.

**Conclusion:**

The patterns of care and treatment outcomes were potentially consistent with world standards, needing future validation. This is the largest study of newly diagnosed nasopharyngeal cancer in Indonesia, a huge disease burden, providing an important basis for the clinical management of this disease.

## Introduction

More than 80,000 cases of nasopharyngeal cancer are reported worldwide each year (Chua et al., 2016). Southeast Asia is endemic for nasopharyngeal cancer, as more than 71% of new cases originate in this area (Chua et al., 2016). Indonesia has the largest population (2.64 million in 2017) among the countries in Southeast Asia, where nasopharyngeal cancer has the fifth highest incidence among malignant neoplasms (UICC New Global Cancer Data of GLOBOCAN, 2018). Therefore, defining the patterns of care and treatment outcomes of patients with nasopharyngeal cancer in Indonesia is important to improve the outcome of this disease worldwide. However, there are few reports on the outcomes of patients with nasopharyngeal cancer treated in Indonesia (Adham et al., 2014; Stoker et al., 2016; Wildeman et al., 2013). Furthermore, the available studies are based on a small sample size, and they may not reflect the actual clinical situation in the country. The aim of the present study was to elucidate the patterns of care and treatment outcomes of patients with nasopharyngeal cancer in Indonesia. To this end, we performed a retrospective study that enrolled the largest number of patients with newly diagnosed nasopharyngeal cancer from Indonesia reported to date.

## Materials and Methods

*Patients and Methods*

*Ethics*

This study was approved by the institutional ethics committee of Dr. Cipto Mangunkusumo National General Hospital (RSCM: Jakarta, Indonesia). This study was exempted from acquisition of written consent for publication from participants by the institutional ethics committee because of its retrospective and observational nature. The study was conducted in accordance with the ethical principles of the Declaration of Helsinki. The anonymity of the patients was preserved.

*Data collection*

Medical records from RSCM between 2012 and 2015 were retrospectively reviewed, and patients with newly-diagnosed and pathologically-confirmed nasopharyngeal cancer who were treated at RSCM were identified. Patients were enrolled if they had available data on age, gender, symptoms, therapeutic regimen, and survival. Stage was determined based on the TNM classification of the Union for International Cancer Control (7^th^ edition) (Sobin et al., 2009).

*Statistical analysis*

Overall survival (OS) was estimated using the Kaplan–Meier method. The differences in OS among subgroups were examined by the log-rank test. All statistical analyses were performed using Prism7 (GraphPad Software, CA, USA). A P-value <0.05 was considered statistically significant.

## Results

*Patient characteristics*

**Figure 1:**
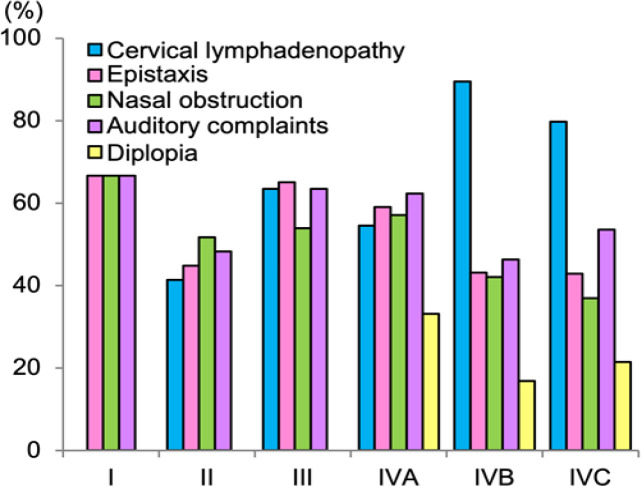
Symptoms at the Time of Diagnosis According to Stage

The retrospective chart review identified 428 patients with newly diagnosed nasopharyngeal cancer who met the inclusion criteria. The median follow-up period was 16 months (range, 1–61 months). The characteristics of the patients are summarized in Table 1 and Supplementary Figure 1. The age peaked at 40–60 years. The ratio of men to women was 2.78. The pathological subtype based on World Health Organization (WHO) criteria (Shanmugaratnam et al., 1991) was III in most cases (92.5%). The majority of patients had advanced disease (stage IV, 77.8%). Patients exhibited various symptoms at the time of diagnosis (Figure 1). Epistaxis, nasal obstruction, and auditory complaints were observed regardless of stage, whereas diplopia and cervical lymphadenopathy were more frequent in patients with stage IV disease.

*Patterns of care*

**Figure 2:**
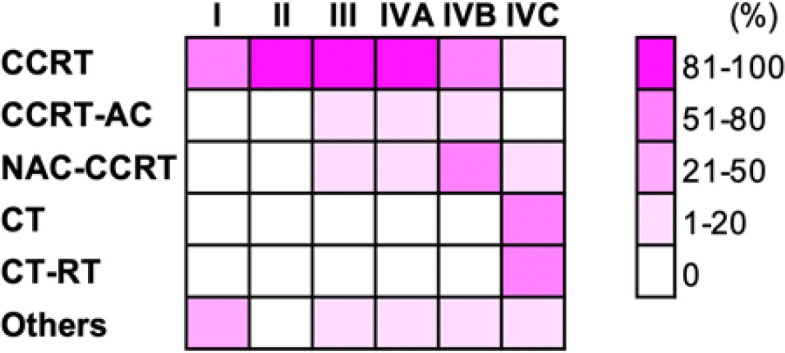
Therapeutic Regimens According to Stage. CCRT, concurrent chemo-radiotherapy; AC, adjuvant chemotherapy; NAC, neoadjuvant chemotherapy; CT, chemotherapy; RT, radiotherapy. Others include RT alone (n = 1, 2, and 1 for stages I, IVA, and IVC, respectively) and NAC-CCRT-AC (n = 1 for each stage III and IVB).

The therapeutic regimens of patients are summarized in Figure 2. Concurrent chemo-radiotherapy (CCRT) was the predominant treatment in patients with stages I–IVB. Neoadjuvant chemotherapy (NAC) was added to CCRT in 3%, 3%, and 43% of patients with stage III, IVA, and IVB, respectively. Adjuvant chemotherapy (AC) was added to CCRT in 13%, 14%, and 6% of patients with stage III, IVA, and IVB, respectively. Chemotherapy was the first choice of treatment in most (81%) patients with stage IVC, followed by radiotherapy in 48% of stage IVC patients. Radiotherapy modalities included two-dimensional radiotherapy (2DRT) (n = 250), three-dimensional conformal radiotherapy (3DCRT) (n = 60), and intensity-modulated radiation therapy (IMRT) (n = 118). 2DRT was administered as follows: (i) lateral opposed fields that cover the primary tumor and the upper cervical lymph node regions for 66–70 Gy in 33–35 fractions, and (ii) 50 Gy in 25 fractions delivered to the lower cervical and supraclavicular lymph node regions. 3DCRT was administered as follows: (i) 70 Gy in 35 fractions delivered to the primary tumor, entire nasopharynx, involved nodes, and nodes at high risk for micrometastasis, and (ii) 50 Gy in 25 fractions delivered to the supraclavicular lymph node region. IMRT was administered as follows: (i) 70 Gy in 33 fractions delivered to the primary tumor and involved nodes; (ii) 60 Gy in 33 fractions delivered to the entire nasopharynx and the node regions at high risk for micrometastasis; and (iii) 54 Gy in 33 fractions delivered to the supraclavicular lymph node region. Chemotherapy consisted of cisplatin, which was administered weekly at 40 mg/m^2^ for CCRT; tri-weekly at 100 mg/m^2^ three to six times for NAC; and tri-weekly at 100 mg/m^2^ three times for AC.

*Treatment and outcomes*

**Figure 3:**
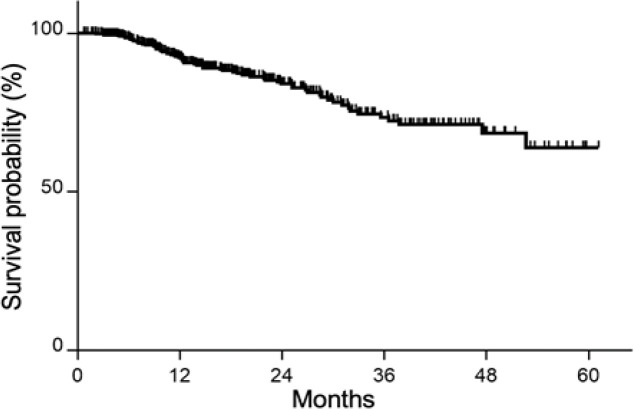
Kaplan–Meier Estimates of Overall Survival for All Patients

**Figure 4:**
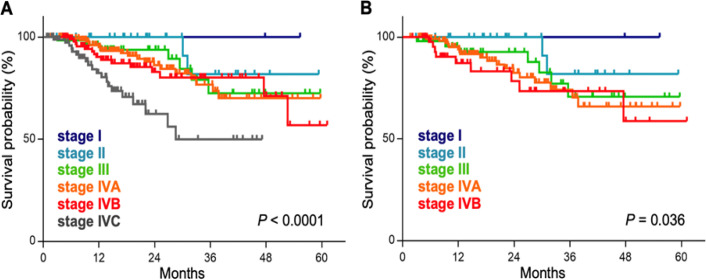
Kaplan–Meier Estimates of Overall Survival According to Stage. (A), All patients; (B), patients treated by concurrent chemo-radiotherapy. P-values were calculated with the log-rank test

For all patients, the 1 and 2 year OS rates were 92.2% and 84.0%, respectively (Figure 3). For all patients according to stage, the 2 year OS rates were 100.0%, 100.0%, 93.8%, 86.2%, 82.9%, and 62.4% for stages I, II, III, IVA, IVB, and IVC, respectively (Figure 4A). The 2 year OS rates of patients treated with CCRT according to stage were 100.0%, 100.0%, 92.6%, 82.4%, and 78.3% for stages I, II, III, IVA, and IVB, respectively (Figure 4B). There was a significant trend toward better OS for early-stage patients in the entire cohort and in the CCRT group (P <0.0001 and <0.036, respectively).

**Figure 5:**
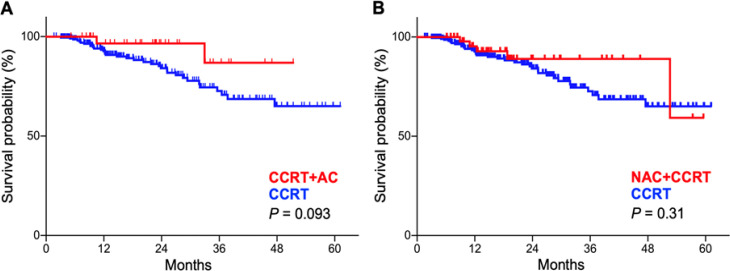
Kaplan–Meier Estimates of Overall Survival for Stages III–IVB Patients. (A), Concurrent chemo-radiotherapy (CCRT) alone vs. CCRT with adjuvant chemotherapy (AC); (B), CCRT alone vs. CCRT with neoadjuvant chemotherapy (NAC). P-values were calculated with the log-rank test

OS was better in patients with stages III–IVB treated with CCRT plus AC than in those treated with CCRT alone, although the difference did not reach statistical significance (P = 0.093) (Figure 5A). There was no significant difference in OS between stages III and IVB patients treated with NAC plus CCRT and those treated with CCRT alone (P = 0.093) (Figure 5B). Classification of patients according to WHO criteria and according to the working formulation (WF) for the malignancy of nasopharyngeal carcinoma proposed by Hsu et al., (1987) did not result in any differences in OS (Supplementary Figure 2). This was probably due to the small number of subgroups (i.e., type I and II for WHO classification, and low-grade and high-grade groups for WF), which may lead to insufficient statistical power.

**Table 1:** Patient Characteristics (n = 428)

Characteristics		Number	%
Age	<20	24	5.6
	20–39	107	25
	40–59	242	56.5
	60–79	55	12.9
Gender	Male	315	73.6
	Female	113	26.4
WHO	I	5	1.2
	II	23	5.4
	III	396	92.5
	NA	4	0.9
WF	High-grade	4	0.9
	Intermediate	393	91.8
	Low-grade	4	0.9
	NA	27	6.4
Stage	I	3	0.7
	II	29	6.8
	III	63	14.7
	IVA	154	36
	IVB	96	22.4
	IVC	83	19.4
T	T1	28	6.5
	T2	65	15.2
	T3	62	14.5
	T4	273	63.8
N	N0	39	9.1
	N1	71	16.6
	N2	170	39.7
	N3	148	34.6
M	M0	345	80.1
	M1	83	19.9

WHO, World Health Organization pathological subtypes; WF, the working formulation for the malignancy of nasopharyngeal carcinoma proposed by Hsu et al. (1987); T, N, and M classifications and stage are based on the criteria of the Union for International Cancer Control (7^th^ edition). NA, not available.

## Discussion

A retrospective review of medical records was performed to analyze the patterns of care and treatment outcomes of 428 patients with newly diagnosed nasopharyngeal cancer. To the best of our knowledge, this is the largest study investigating the treatment of nasopharyngeal cancer in Indonesia. The patient demographics of the study regarding age, male-to-female ratio, and WHO subtypes were consistent with the typical patient demographics for nasopharyngeal cancer arising in Southeast Asia (Chua et al., 2016). A significant trend toward better OS was observed for all patients and for CCRT-treated patients. These observations indicate the unbiased enrollment of patients in this study and the robustness of the medical chart review. The results of the present study provide an important basis for the clinical management of nasopharyngeal cancer in Southeast Asia.

Several studies reported the treatment outcomes of patients with nasopharyngeal cancer in Indonesia (Adham et al., 2014; Stoker et al., 2016; Wildeman et al., 2013). Stoker et al., (2016) retrospectively investigated the outcomes of 142 nasopharyngeal cancer patients treated with radiotherapy with or without chemotherapy with curative intent. The 2 year OS was 58% for this cohort that predominantly contained stages III–IVB patients. Wildeman et al., (2013) retrospectively investigated the outcomes of 78 nasopharyngeal cancer patients receiving radiotherapy with or without chemotherapy with curative intent. Based on Figure 1 of their paper, the 2 year OS was approximately 50% for this cohort that predominantly contained stage IIII–IVB patients. Adham et al. (2014) retrospectively investigated the outcomes of 42 young (i.e., <31 years old) patients with nasopharyngeal cancer treated with radiotherapy with or without chemotherapy with curative intent. In this cohort that predominantly contained stages III–IVB patients, the 2 year OS was 39–71%, including patients who did not develop distant metastasis. These studies reported the outcomes for a radiotherapy institute in Yogyakarta, Indonesia, which covers three provinces (i.e., Yogyakarta, Central Java, and East Java) and includes a population of approximately 70 million. The large number of patients treated in this facility combined with the geographical difficulty associated with accessing the institute contributes to its long treatment-waiting times, which can cause cancer progression. Therefore, these results may not be generalized as the clinical outcomes of patients with nasopharyngeal cancer treated in Indonesia. Furthermore, insufficient sample numbers decrease the scientific validity of these results.

The treatment outcomes of the present study look more favorable compared with the previous studies, and closer to the world standards presented in a review article (Chua et al., 2016). The patients treated in RSCM come from Jakarta area (30%), greater Java area (30%), and outside Java (40%). From this perspective, it can be said that the patient demographic at RSCM analyzed in the present study represents national demographic structure of Indonesia. Together, the results of the present study indicate that this country is potentially capable of providing standard-level care for nasopharyngeal cancer. However, the waiting time for radiotherapy can be long for certain patients in our institution, as well as for those receiving NAC. Validation of the outcomes of this study is warranted using a prospective cohort in the future. In particular, the benefit of adding AC to CCRT for advanced disease, which was demonstrated as marginal in this study, should be further investigated because it is a controversial issue worldwide (Chua et al., 2016). In addition, the study is limited by the lack of clinical information such as disease-free survival, treatment-related toxicities, and completion rate of treatment.

Short follow up period (median of 16 months) should be noted as the limitation of this study. As the national referral hospital, RSCM accepts patients from all over Indonesia. Due to maintain the capacity of RSCM, the patients originating from other islands or provinces are followed up by the hospitals in the area. For such cases, follow up for this study was conducted using phone call; however, the follow up was technically difficult (e.g., invalid phone number) in a portion of participants. Construction of better nationwide follow up systems should be considered in future.

In summary, we provided the largest scaled data reported to date on the patterns of care and treatment outcomes of patients with newly diagnosed nasopharyngeal cancer in Indonesia, an endemic country for this disease. The patterns of care and treatment outcomes observed in this retrospective single institution study were potentially in line with world standards, although it is difficult to draw a solid conclusion due to short follow-up. These data provide an important basis for the clinical management of nasopharyngeal cancer in Southeast Asia.
